# Macular hole following contusion syndrome

**DOI:** 10.11604/pamj.2015.21.71.4213

**Published:** 2015-05-29

**Authors:** Belhassan Salim, Daoudi Rajae

**Affiliations:** 1 University of Mohamed V Souissi, Hopsital of Specialities, Departement of Ophtalmology A, Rabat, Morroco

**Keywords:** Macular, ophthalmology, eye

## Image in medicine

A young 15 year old boy was brought to the emergency department of ophthalmology after he have had an acute accident during a tennis game, the ball accidently heat him in her left eye resulting palpebral edema and decreased visual acuity. In the emergency room, the ophthalmic exam found visual acuity in the left eye at 1/20 and loss of near acuity, 10/10 in the right eye and anterior segment unremarkable. The fundus of the eye reached found a macular hole well defined with diffuse berlin edema (A). The OCT a 489 micrometer stage 3 macular hole (B). The patient was placed in short-term corticoid treatment and controlled one week later, the visual acuity rises to 2/10. One month later a regular exam demonstrate a stable visual acuity at 2/10 but a much bigger macular hole on the fundus (C) and respectively in the OCT the diameter rises to 1155 micrometer, stage 4 (D). Macular holes can be treated with surgery through a procedure called a vitrectomy. The operation is done to remove the vitreous jelly and additional tissue that may be keeping the macular hole open. At surgery, the eye is filled with a special gas that stays in the eye and slowly dissolves over a few weeks. The gas forms an air bubble that gently presses on the hole and encourages new tissue to close the hole. Non-treated macular holes stabilize with vision of about 20/200.

**Figure 1:**
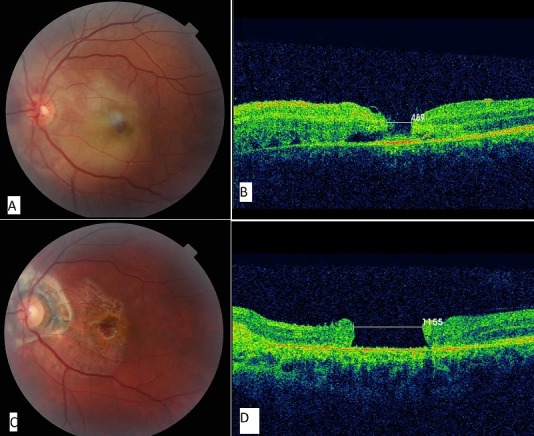
A) macular hole on the fundus at day 1 of contusion trauma; B) corrensponding OCT at day 1 of trauma showing a stage 3 macular hole; C) macular hole evolution after one month; D) the macular hole got bigger one month later: stage 4

